# Measuring Learning of Medical Students through ‘Programmatic Assessment'

**DOI:** 10.12669/pjms.341.14606

**Published:** 2018

**Authors:** Rehan Ahmed Khan

**Affiliations:** Prof. Dr. Rehan Ahmed Khan, Professor of Surgery and Assistant Dean Medical Education, Islamic International Medical College, Riphah International University, Islamabad, Pakistan. Email: surgeonrehan@gmail.com

Assessment is measurement of learning. It determines the level of competence.^1^ If assessment of a medical student is faulty, it would lead to the production of an incompetent doctor, who will be a threat for the community. Hence it is important to have an assessment system in place, which effectively measures the learning of a student.

The prevalent assessment system in Pakistan is based on internal and external assessment in majority of the medical colleges.^2^ National Accreditation and regulatory body's medical curriculum^3^ provides medical colleges with guideline of using 10% internal and 90% external assessment. Internal assessment constitutes of class tests with varying contents from different subjects, conducted by the medical college whereas the external assessment is the ‘end of year' assessment or the ‘Professional examination', conducted by the University. ‘Assessment drives learning'^4^, hence this system of assessment encourages student to study for examination at the end of year because of the 90% percentage given to the professional examination. The reward for the student who studies for the whole academic year is only 10%, thus discouraging the continuous learning process. Another major flaw in this system is that only one data point i.e. ‘Professional Examination' mainly decides the pass/fail decision of the student.

The science of assessment is no more mere ‘internal' or ‘external'. It has even moved beyond using only the simple concepts of ‘Formative' and ‘Summative' assessments to ‘Programmatic Assessment'.^5^,^6^ Formative assessment is assessment for learning and summative assessment is assessment of learning. In the current system, summative assessment comprises of 10% internal and 90% external assessment, which decides the pass/fail decision whereas formative assessment (continuous assessment) is completely at the discretion of the medical colleges. Formatives assessment plays a major role in providing timely feedback to the student, hence providing them with an opportunity to improve their grades and increase their learning.^7^,^8^ However, when formatives have no clear weightage assigned to them, majority of the students do not attend these assessments.

Cees Van der Leuten describes ‘Programmatic assessment'^5^,^6^ as an assessment system which comprises of low, mid and high stakes assessment conducted throughout the academic year. All these assessments have a weightage and are made of several data points (Examinations). Hence student pass/fail decision does not rely on a single data point but on sum of multiple data points. Low stake and mid stake assessment are done as continuous assessments i.e throughout the academic year and have both formative and summative component. Students in these assessments get qualitative and quantitative results in the form of verbal/written feedback and numbers or grades. High stake assessment is then taken at the ‘End of year' as the Professional exam and has major summative component. This system of assessment encourages the students to study throughout the year as their pass/fail decision depends on aggregate of multiple data points. This also provides them with multiple opportunities to improve their learning as they get feedback throughout the year.

In our present system, this can be easily employed by giving medical colleges a share of at least 50% of assessment comprising of low and mid stake assessments; low stakes being 10% and mid stakes 40%. Low stakes assessment can comprise of the class tests with major emphasis on formative assessment i.e giving students timely and detailed qualitative feedback whereas Mid stakes assessments can be taken in the middle and towards the end of the academic year comprising of larger content from the syllabus.

Perhaps in the past, the main concern of giving medical college an equal share of assessment was the question of transparency of assessment, leading to the system of 10% and 90% distribution of internal and external assessment respectively. However, in our present scenario this may not stand valid as many universities now have constituent and affiliated medical colleges and also medical colleges are now under the umbrella of Medical universities as opposed to non-medical university in the past. It is important to mention here that World Federation for Medical Education^9^ (WFME) and ASPIRE^10^ initiatives for achieving excellence in assessment recommend evaluation of Assessment systems by the accreditation bodies / university. Hence the accreditation body or university can ensure the transparency of the ‘share of internal assessment' given to a medical college enabling medical students to study all year long.

It's high time that we need to modernise our assessment policies, procedures and methods. Assessing students through programmatic assessment using multiple data points to promote a student to next class rather a single data point (carrying 90% weightage) and evaluation of “internal assessment' through external inspectors to ensure its quality, is the need of the hour.
